# Human Breast Milk and Antiretrovirals Dramatically Reduce Oral HIV-1 Transmission in BLT Humanized Mice

**DOI:** 10.1371/journal.ppat.1002732

**Published:** 2012-06-14

**Authors:** Angela Wahl, Michael D. Swanson, Tomonori Nochi, Rikke Olesen, Paul W. Denton, Morgan Chateau, J. Victor Garcia

**Affiliations:** 1 Division of Infectious Diseases, Center for AIDS Research, University of North Carolina at Chapel Hill, School of Medicine, Chapel Hill, North Carolina, United States of America; 2 Department of Infectious Diseases, Aarhus University Hospital, Skejby, Denmark; Emory University, United States of America

## Abstract

Currently, over 15% of new HIV infections occur in children. Breastfeeding is a major contributor to HIV infections in infants. This represents a major paradox in the field because *in vitro*, breast milk has been shown to have a strong inhibitory effect on HIV infectivity. However, this inhibitory effect has never been demonstrated *in vivo*. Here, we address this important paradox using the first humanized mouse model of oral HIV transmission. We established that reconstitution of the oral cavity and upper gastrointestinal (GI) tract of humanized bone marrow/liver/thymus (BLT) mice with human leukocytes, including the human cell types important for mucosal HIV transmission (i.e. dendritic cells, macrophages and CD4^+^ T cells), renders them susceptible to oral transmission of cell-free and cell-associated HIV. Oral transmission of HIV resulted in systemic infection of lymphoid and non-lymphoid tissues that is characterized by the presence of HIV RNA in plasma and a gradual decline of CD4^+^ T cells in peripheral blood. Consistent with infection of the oral cavity, we observed virus shedding into saliva. We then evaluated the role of human breast milk on oral HIV transmission. Our *in vivo* results demonstrate that breast milk has a strong inhibitory effect on oral transmission of both cell-free and cell-associated HIV. Finally, we evaluated the effect of antiretrovirals on oral transmission of HIV. Our results show that systemic antiretrovirals administered prior to exposure can efficiently prevent oral HIV transmission in BLT mice.

## Author Summary

Infected children acquire HIV from their mother *in utero*, intrapartum or by ingesting their mother's breast milk which can contain both HIV particles (cell-free) and HIV-infected cells (cell-associated). Although breastfeeding is attributed to a significant number of HIV infections in children, most breastfed infants remain uninfected despite prolonged and repeated exposure to HIV. This limited transmission has led to two apparently contradictory roles for milk in HIV infection: vector of transmission or vehicle of protection? Milk has a strong inhibitory effect on HIV infection *in vitro*. However, this has never been demonstrated in an *in vivo* system. In the present study, we address this paradox in a bone marrow/liver/thymus (or BLT) humanized mouse model of oral transmission of cell-free and cell-associated HIV. We demonstrate that human breast milk has potent HIV inhibitory activity that can prevent oral transmission of cell-free and cell-associated HIV *in vivo*. Our results provide key insight into oral HIV transmission and the protective role of milk. However, since transmission can and does occur in some instances after continued exposure to HIV in milk, we demonstrate that oral HIV transmission can be efficiently prevented in BLT humanized mice by the systemic administration of antiretrovirals.

## Introduction

Pediatric HIV infection is associated with an accelerated course of disease and high mortality rate. In the absence of antiretroviral therapy, only 65% of HIV-infected children survive until their first birthday and less than half will reach two years of age [1]. Most children acquire HIV from their mother *in utero*, intrapartum or orally during breastfeeding [2]. In developed countries the incidence of mother-to-child transmission of HIV is extremely low; HIV-infected women receive antiretroviral therapy during pregnancy and delivery and abstain from breastfeeding. Furthermore, their children receive antiretroviral prophylaxis at birth and for several weeks thereafter. The majority of HIV-infected children live in sub-Saharan Africa where HIV-positive women have limited access to antiretroviral drugs and the health benefits of breastfeeding outweigh the risk of HIV transmission [3].

Despite the presence of innate factors in human breast milk that display strong HIV inhibitory activity *in vitro* [4]–[12], up to 44% of HIV infections in children can be attributed to breastfeeding. The risk of acquiring HIV after a single day of breastfeeding is extremely low (0.00028 per day of breastfeeding) [13], however, after ingesting liters of breast milk over a span of several months to years (∼250 liters per year), 5–20% of infants born to HIV-infected women will eventually become infected with HIV in the absence of any preventative measures [14]. Exclusive breastfeeding (not allowing any water, juice or solid foods) has been associated with a drastic decrease in the HIV transmission rate through breastfeeding, indicating that breast milk acts as a vehicle of protection [14]. However, elevated levels of HIV particles (cell-free virus) and HIV-infected cells (cell-associated virus) in breast milk of HIV-positive women are associated with an increased risk for HIV transmission during breastfeeding [15]–[17]. Although it has been reported that a 10-fold increase in cell-free or cell-associated HIV in breast milk is associated with a 3-fold increase in transmission [15], it is still unclear whether cell-free and/or cell-associated virus are transmitted during breastfeeding. Furthermore, it is not known if the frequency of cell-free and cell-associated HIV transmission varies at different stages of lactation (i.e. colostrum, early breast milk and mature breast milk). Therefore, successful interventions may need to prevent transmission of both cell-free and cell-associated HIV during breastfeeding by reducing the viral load and number of infected cells in breast milk and/or by directly inhibiting infection of the infant oral and GI mucosa.

The development of effective strategies to prevent HIV acquisition during breastfeeding would be significantly enhanced by a small animal model of oral HIV infection that could be utilized to study the relative contribution of cell-free and cell-associated virus in transmission, the mechanism for oral transmission of cell-free and cell-associated HIV and the innate HIV inhibitory activity of human breast milk. In addition, animal models are needed to test the efficacy of novel approaches to prevent transmission of cell-free and cell-associated HIV in breast milk. Thus, we developed an oral HIV transmission model based on BLT humanized mice [18]. We then used this model to demonstrate 1) efficient cell-free and cell-associated oral HIV transmission, 2) transmission can occur in the oral cavity or the upper GI tract, 3) transmission can be prevented with antivirals and 4) that human breast milk dramatically reduces oral HIV transmission.

## Results

BLT humanized mice are created by transplanting autologous fetal liver-derived human CD34^+^ hematopoietic progenitor cells into mice previously implanted with a piece of human fetal liver sandwiched between two small pieces of human fetal thymus. Humanized BLT mice show robust reconstitution with virtually all human hematopoietic cell types that are present in primary, secondary and effector immune organs. The immune cells present in BLT humanized mice have been shown to be able to mount both humoral and cellular immune responses to model antigens and viruses. Our laboratory has further established that the systemic reconstitution with human hematopoietic cells renders BLT humanized mice susceptible to parenteral, rectal and vaginal HIV infection [19]–[24]. More recently, we also demonstrated that BLT mice can be utilized to assess the efficacy of pre- and post-exposure antiretroviral prophylactic strategies to prevent mucosal and parenteral HIV infection [19]–[22].

### Reconstitution of the oral cavity of BLT mice with human hematopoietic cells

Highlighting the importance of the oral cavity as the first site of exposure for HIV that is transmitted during breastfeeding, our initial aim for this study was to examine if the oral mucosa of BLT mice is repopulated with human hematopoietic cells. For this purpose, we used immunohistochemistry (IHC) analysis. Specifically, we determined whether the oral mucosa of BLT mice is reconstituted with human hematopoietic (CD45^+^) cells that include the types of cells known to play an essential role in mucosal HIV transmission: human dendritic cells (CD11c^+^), macrophages (CD68^+^), B cells (CD20^+^) and T cells (CD3^+^).

**Figure 1:**
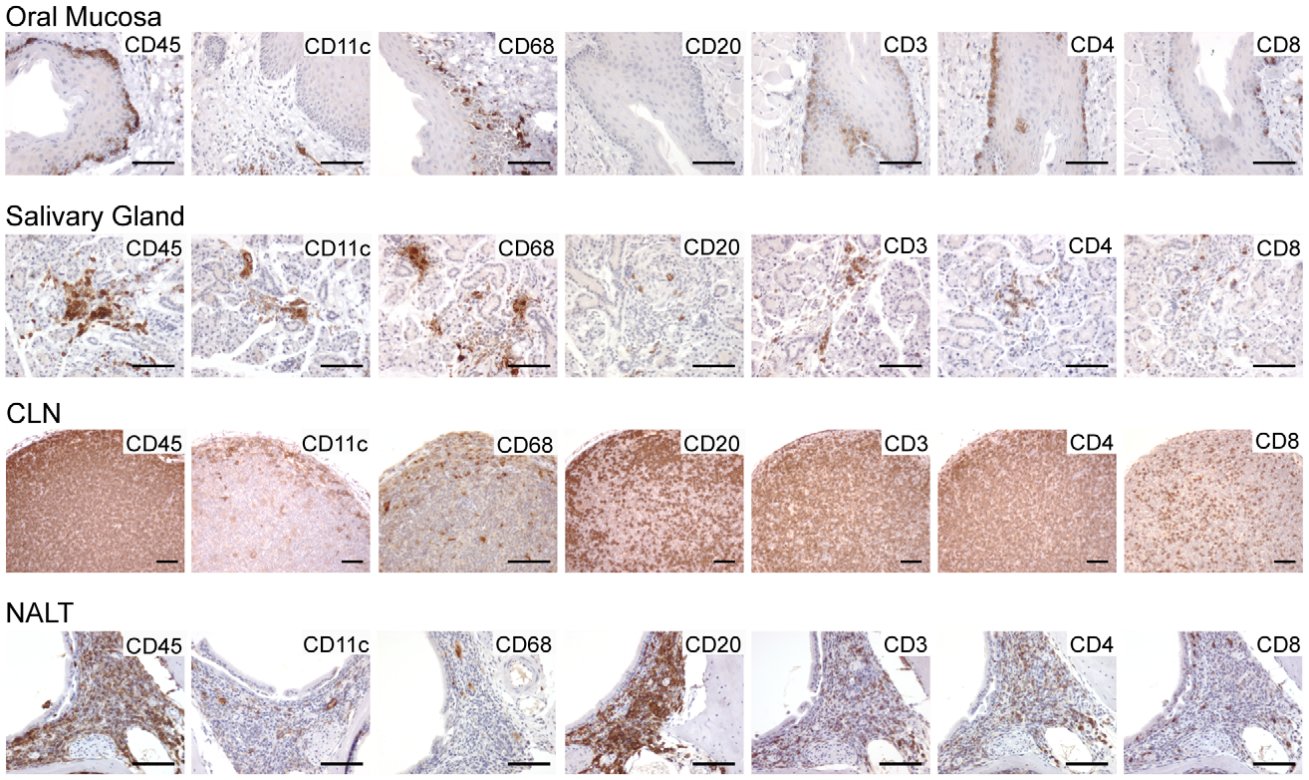
Reconstitution of the oral cavity of humanized BLT mice with human hematopoietic cells. The oral mucosa, salivary glands, CLN and NALT were harvested from BLT mice and stained with the appropriate antibodies to verify the presence of human leukocytes (CD45^+^) including dendritic cells (CD11c^+^), macrophages (CD68^+^), B cells (CD20^+^), T cells (CD3^+^), CD4^+^ T cells (CD4^+^) and CD8^+^ T cells (CD8^+^). Positive cells appear brown. Scale bars = 100 µm.

In the oral mucosa of BLT mice, human macrophages were detected in the epithelial layer, near the basement membrane, and in the lamina propria whereas human dendritic cells were predominantly present in the lamina propria. Human CD4^+^ and CD8^+^ T cells were also present in the epithelial layer, appearing as a band at the basement membrane, and in the underlying lamina propria. In contrast, IHC analysis did not detect the presence of human B cells in either the epithelium or lamina propria in the oral mucosa of BLT mice (Figure 1). While comparable studies in humans are limited, macrophages, dendritic cells and T cells have also been identified in the oral mucosa of infants [25]. In addition, although the presence of B cells in the oral mucosa of infants has not been investigated, our data demonstrating a lack of B cells in the oral mucosa of BLT mice is consistent with what has been described in human adults [26]. All together, these results indicate that the oral mucosa of BLT mice, like infants, possesses human macrophages, dendritic cells and T cells, the human cell types important for HIV transmission.

Next, we used IHC to determine if human hematopoietic cells are present in the salivary glands and lymphoid tissues proximal to the oral mucosa of BLT humanized mice, as these tissues may serve as early sites for viral dissemination. The salivary glands are directly connected to the oral mucosa by excretory ducts that secrete saliva into the oral cavity. In infected humans, HIV-infected cells have been identified in the salivary glands and HIV RNA has been detected in saliva [27]–[29]. IHC analysis revealed that the salivary glands of BLT mice are reconstituted with human target cells for HIV infection (macrophages, dendritic cells and CD4^+^ T cells) as wells as human CD8^+^ T cells and B cells (Figure 1). The cervical lymph nodes (CLN), which drain the oral mucosa, and the nasal-associated lymphoid tissue (NALT) of BLT mice were also repopulated with human macrophages, dendritic cells, T cells and B cells (Figure 1). Collectively, these data demonstrate that the oral cavity of BLT mice is reconstituted with human hematopoietic cells including all of the human cell types important for HIV transmission and dissemination (i.e. dendritic cells, macrophages and CD4^+^ T cells).

### Identification of HIV target cells in the upper GI tract of BLT mice

**Figure 2:**
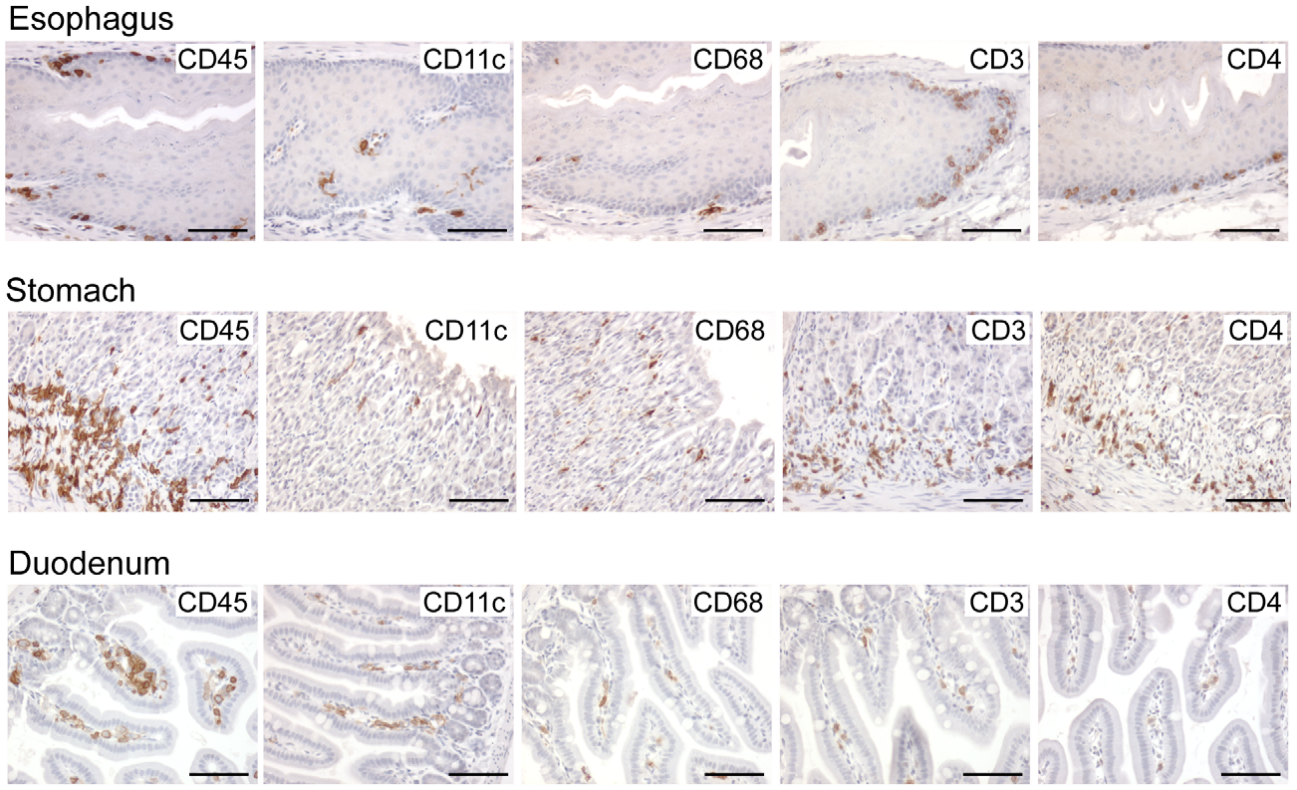
The upper GI tract of humanized BLT mice is repopulated with HIV target cells. The esophagus, stomach and duodenum were harvested from BLT mice for IHC analysis to determine if these potential sites for HIV transmission following an oral exposure possess HIV target cells. The tissues harvested were stained with the appropriate antibodies to verify the presence of human leukocytes (CD45^+^) including dendritic cells (CD11c^+^), macrophages (CD68^+^) and T cells (CD3^+^), specifically, CD4^+^ T cells (CD4^+^). Positive cells appear brown. Scale bars = 100 µm.

Although the oral mucosa is the first surface exposed to HIV that is transmitted through breastfeeding, it is not clear if transmission occurs in the oral cavity or in the upper GI tract of infants. HIV is typically inactivated in acidic environments like the stomach but the pH of the infant stomach is considerably higher than that of adults [30] which may allow transmission to occur in the upper GI tract. Therefore, we used IHC to determine if the human cell types important for HIV transmission are present in the esophagus, stomach and upper small intestine of BLT mice. IHC analysis demonstrated the presence of CD45^+^ human cells in the esophagus, stomach and duodenum of BLT mice. Specifically, human dendritic cells, macrophages and CD4^+^ T cells were all present in these tissues (Figure 2). We also identified a dense population of human hematopoietic cells at the gastroesophageal (GE) junction, where the esophagus joins the stomach (Figure S1).

In regard to the relative distribution of HIV target cells in the upper GI tract, in the esophagus of BLT mice, human macrophages, dendritic cells and CD4^+^ T cells were located in the basal layer of the epithelium and in the lamina propria (Figure 2). Human dendritic cells, macrophages and CD4^+^ T cells were also identified in the epithelium and lamina propria of the stomach as well as the lamina propria of the duodenum (Figure 2). These findings are consistent with previous reports which demonstrated reconstitution of the gastrointestinal tract of BLT mice with human target cells for HIV infection [23], [31]. Although the presence and distribution of these immune cell types in the esophagus, stomach and upper intestine of human infants has not been systematically explored, macrophages, dendritic cells and CD4^+^ T cells have been identified in the upper GI tract of healthy adults [29], [32]–[36]. All together, these results demonstrate robust reconstitution of the upper GI tract of BLT mice with human cells highlighting the potential for transmission to occur at these important mucosal sites.

### Oral transmission of HIV in BLT humanized mice

**Figure 3:**
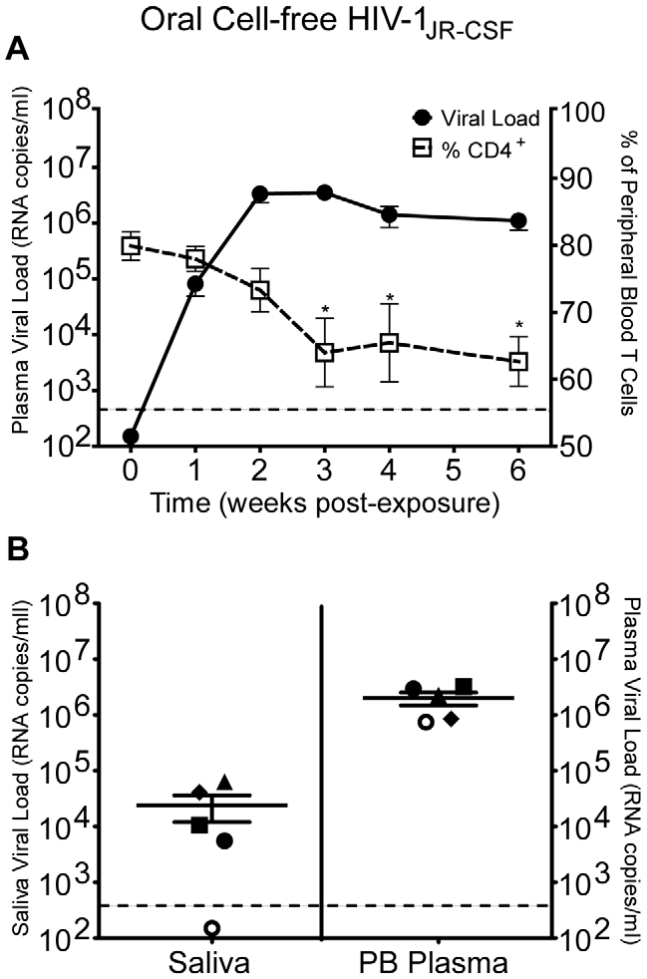
Oral transmission of cell-free HIV in humanized BLT mice. (A) BLT mice (n = 10) were exposed orally to the CCR5-tropic HIV-1 isolate JR-CSF. Infection was monitored weekly by measuring the plasma viral load and percentage of human CD4^+^ T cells in peripheral blood. A two-tailed Mann-Whitney U test was used to compare the percentages of CD4^+^ T cells in peripheral blood pre-exposure (week 0) and post-exposure (p values<0.05 are indicated with an asterisk). (B) Saliva and peripheral blood (PB) were collected from five BLT mice 5–8 weeks following oral HIV exposure. Saliva was collected on the same day as peripheral blood or one week later. The corresponding saliva and peripheral blood viral loads for each mouse are shown with the same shape. The limit of detection for the assay is illustrated with a dashed line.

Once we established the presence of human target cells for HIV infection in the oral cavity and upper GI tract of BLT mice, we proceeded to determine if BLT mice are susceptible to oral HIV infection. For this purpose, we exposed BLT mice to a single dose of cell-free HIV-1_JR-CSF_, a CCR5-tropic isolate, administered directly into the oral cavity. We then monitored infection in peripheral blood by measuring viral load levels essentially as we have previously described [22]. In addition, as a measure of the pathogenic effects of HIV infection, we also monitored human CD4^+^ T cell depletion in peripheral blood. Consistent with the presence of HIV target cells in the oral mucosa and upper GI tract of BLT mice, reproducible oral HIV transmission was observed. Specifically, viral RNA was readily detected in the plasma of all BLT mice exposed orally to HIV-1_JR-CSF_ (Figure 3A). The presence of viral RNA in the plasma of infected mice preceded a significant decrease in the percentage of CD4^+^ T cells in the peripheral blood that became evident at three weeks post-exposure (Figure 3A).

Once infection was confirmed in the plasma of these mice, we also determined the presence of viral RNA in saliva. Our results demonstrate the presence of HIV RNA in the saliva of 4/5 infected BLT mice examined. Consistent with what is observed in humans, the viral load in the saliva of each BLT mouse was lower than the viral load in plasma (Figure 3B) [27], [28]. The presence of viral RNA in the saliva of BLT mice is consistent with the productive infection of human hematopoietic cells within the oral mucosa and/or salivary glands of BLT mice. Taken together, these findings demonstrate BLT humanized mice are susceptible to oral HIV transmission that results in systemic infection, as demonstrated by the presence of HIV RNA in plasma and saliva.

### Transmission of HIV via the upper GI tract in BLT mice

**Figure 4:**
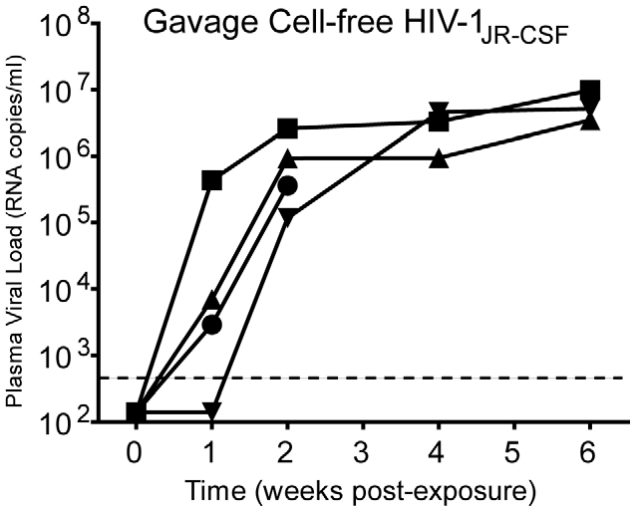
Susceptibility of BLT mice to HIV infection after administration via gavage. To determine if the upper GI tract of BLT mice is susceptible to HIV transmission, we evaluated HIV acquisition after a single direct administration of virus to the upper GI tract via gavage. Infection was monitored in peripheral blood by determining the levels of viral load in BLT mice (n = 4) receiving cell-free HIV-1_JR-CSF_ directly into the stomach by gavage. The viral load (RNA copies/ml) for each mouse is indicated and the limit of detection for the assay is illustrated with a dashed line.

Following ingestion of breast milk from HIV-infected mothers, HIV transmission to neonates may occur in the oral cavity and/or upper GI tract. In BLT humanized mice, this could also be the case given the robust reconstitution of the oral cavity and upper GI tract with human dendritic cells, macrophages and CD4^+^ T cells. In order to determine if HIV transmission can occur distal to the esophagus, we introduced HIV-1_JR-CSF_ directly into the stomach of BLT mice by oral gavage. After exposure by gavage, viral RNA could be readily detected in the plasma of all BLT mice two weeks post-exposure (Figure 4). This finding indicates that the mucosal surfaces of BLT mice distal to the oral cavity can be directly infected with HIV to effect transmission. These results serve as evidence for this mode of HIV transmission.

### Systemic dissemination of HIV infection following oral exposure

**Figure 5:**
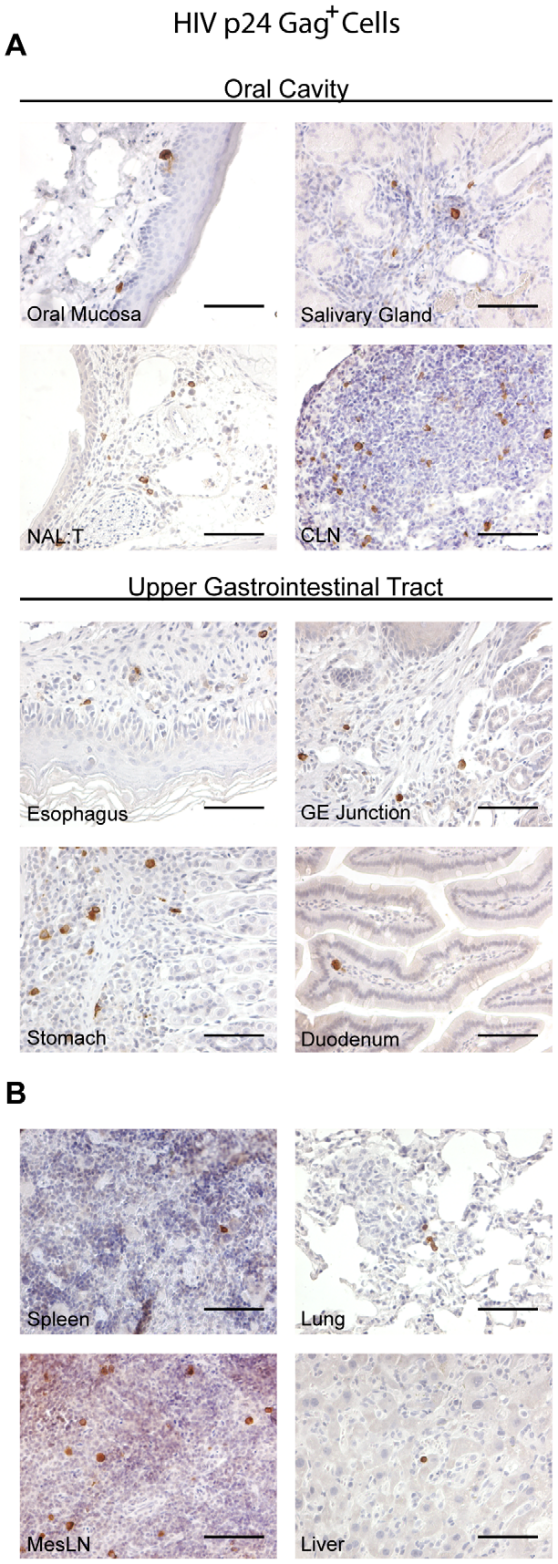
Infection of the oral cavity and upper GI tract following oral HIV transmission. (A) Tissues were harvested from the oral cavity and upper GI tract of infected BLT mice following oral HIV exposure and stained with an antibody directed against HIV p24 Gag. (B) HIV p24 Gag staining also identified infected cells in peripheral lymphoid, mucosal and non-mucosal tissues isolated from infected BLT mice outside of the oral cavity and upper GI tract. HIV-infected cells appear brown. Scale bars = 100 µm.

In humans, once HIV establishes an infection, lymphoid organs become the primary site of viral replication which subsequently allows HIV to disseminate to distal tissues resulting in a systemic infection [37]. In order to evaluate the systemic nature of the infection that occurs after oral exposure, we harvested tissues from infected BLT mice 2–8 weeks post-exposure. IHC analysis demonstrated the presence of productively infected (HIV p24 Gag^+^) cells in the mucosal tissues of the oral cavity and upper GI tract of BLT mice. We observed infected cells in the oral mucosa and salivary glands of BLT mice as wells as in proximal lymphoid tissues (CLN and NALT) (Figure 5A). HIV-infected cells were also identified in the esophagus, stomach and duodenum of BLT mice following oral transmission (Figure 5A). Substantiating the role of lymphoid tissues as the principal site for productive virus infection, HIV-infected cells were detected in the spleen and lymph nodes of infected BLT mice (Figure 5B). Furthermore, we observed viral dissemination into non-lymphoid tissues including the lung and liver (Figure 5B). These results demonstrate systemic infection of BLT mice following oral HIV transmission and highlight some of the remarkable similarities in HIV infection between humans and BLT mice.

### Susceptibility of BLT mice to HIV-1 transmitted/founder viruses

During mucosal transmission, HIV encounters several physical and immunologic barriers that allow only one to a few variants from a diverse donor pool to cross the mucosa and establish systemic infection (reviewed in [38], [39]). A better understanding of the molecular and biological determinants that bestow a selective advantage for mucosal transmission of these transmitted/founder (T/F) viruses will aid the development of novel vaccines and other prevention strategies. Hence, our next objective was to determine if CCR5-tropic T/F viruses can establish a systemic infection in BLT mice following an oral exposure.

We exposed groups of BLT mice orally to one of three different T/F viruses: HIV-1_RHPA_, HIV-1_CH040_ or HIV-1_CH077_ [40]–[42]. All of these T/F viruses replicate efficiently in activated primary human CD4^+^ T cells *in vitro* [41]. As a control, BLT mice were also exposed to HIV-1_JR-CSF_. In order to appreciate any subtle difference in transmission efficiency between HIV-1_JR-CSF_ and the three T/F viruses, BLT mice were orally administered a single lower dose of HIV (6×10^5^ TCIU). Even at this lower dose, at two weeks post-exposure, viral RNA was detected in the plasma of 75% of BLT mice exposed orally to HIV-1_JR-CSF_. The T/F virus HIV-1_CH040_ was also readily transmitted following oral exposure (100%). Only 66% and 33% of BLT mice orally exposed to HIV-1_RHPA_ and HIV-1_CH077_ respectively became infected (Table 1). These results indicate that BLT mice are susceptible to oral transmission of T/F viruses and that T/F viruses may not be equally capable of oral transmission. Furthermore, these findings demonstrate that BLT mice can be used to evaluate the transmission efficiency of T/F viruses.

**Table 1:** Oral transmission of T/F viruses in BLT mice.

HIV-1 Virus	T/F Virus	Probable Route of Transmission in Humans	Oral Dose (TCIU)	Oral Transmission Efficiency
JR-CSF	N	N/A	0.6×10^6^	75% (3 of 4)
			1.4×10^6^	100% (10 of 10)
RHPA	Y	Vaginal	0.6×10^6^	66% (2 of 3)
CH044	Y	Rectal	0.6×10^6^	100% (4 of 4)
CH077	Y	Rectal	0.6×10^6^	33% (1 of 3)

Transmission is defined by the presence of RNA in the plasma of BLT mice.

### Oral transmission of cell-associated HIV in BLT mice

**Figure 6:**
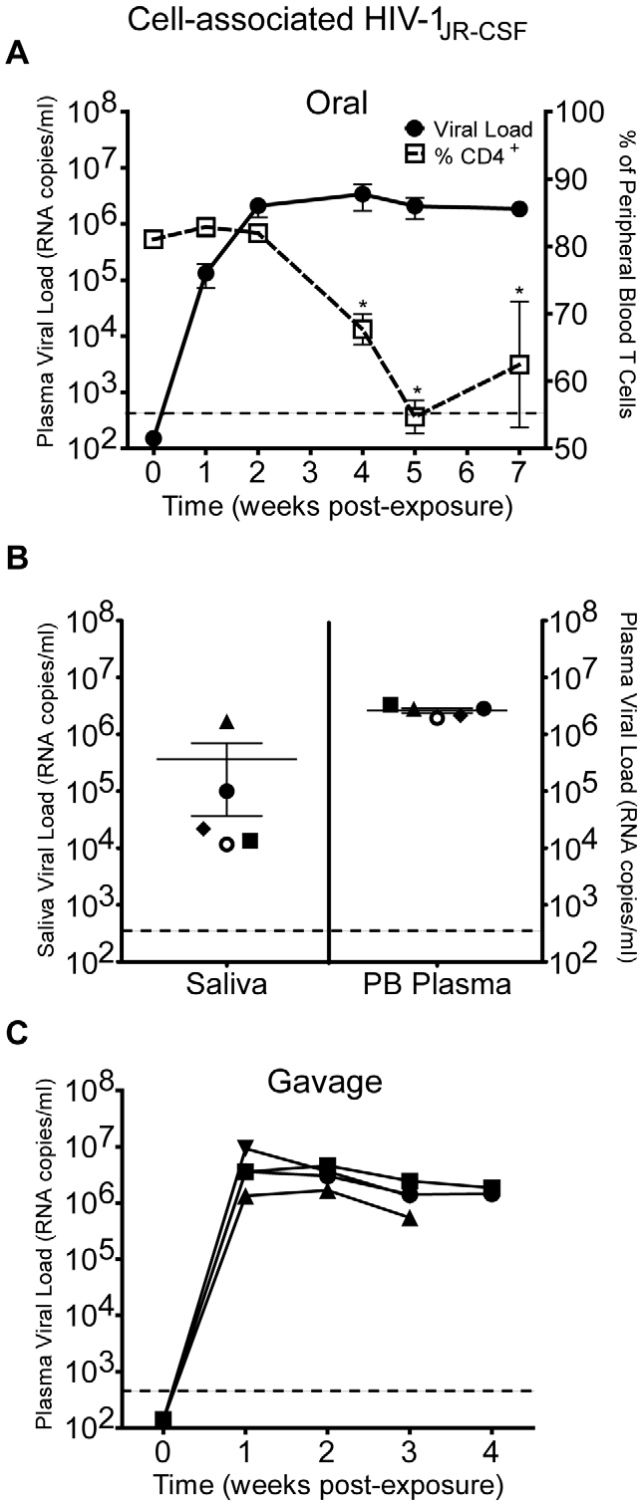
Oral transmission of cell-associated HIV in humanized BLT mice. (A) BLT mice (n = 7) were exposed orally to HIV-1_JR-CSF_ infected PBMCs. Transmission was monitored weekly by measuring the plasma viral load and percentage of human CD4^+^ T cells in peripheral blood. A two-tailed Mann-Whitney U test was used to compare the percentages of CD4^+^ T cells in peripheral blood pre-exposure (week 0) and post-exposure (p values<0.05 are indicated with an asterisk). (B) Saliva and peripheral blood (PB) were harvested from five BLT mice following oral HIV exposure with cell-associated HIV-1_JR-CSF_. Saliva and peripheral blood were collected from BLT mice on the same day and the corresponding saliva and peripheral blood viral loads for each mouse are shown with the same color and shape. (C) HIV transmission of cell associated virus administered via gavage into the stomach of BLT mice. Shown is the viral load in the peripheral blood of BLT mice (n = 4) receiving a single dose of HIV-1_JR-CSF_ infected PBMCs directly into the stomach by gavage.

Cell-free and cell-associated HIV can be detected in the breast milk of HIV-infected women and it is currently unclear if either or both are responsible for the transmission event resulting in infection [15], [43]–[45]. Therefore, once we demonstrated oral transmission of cell-free HIV, we established the oral transmission of cell-associated HIV. For this purpose, we first generated stocks of allogeneic human PBMCs infected with HIV-1_JR-CSF_ *in vitro*. The efficiency of infection and the numbers of productively infected cells in the individual stocks were determined by intracellular staining for HIV p24 Gag and subsequent flow cytometric analysis. BLT mice were then exposed orally to a single dose of HIV-infected cells (3.75×10^5^ HIV Gag p24^+^ cells) and monitored for infection by determining the presence of viral RNA in peripheral blood plasma. We observed 100% transmission by two weeks post-exposure when BLT mice were exposed orally to a single inoculum of HIV-infected cells (Figure 6A). These results demonstrate efficient oral transmission of cell-associated HIV.

Similar to oral exposure to cell-free HIV, following the appearance of viral RNA in plasma, oral transmission of cell-associated HIV was characterized by a significant decrease in the percentage of CD4^+^ T cells in peripheral blood (Figure 6A). Also similar to what we observed after cell-free HIV infection, we readily detected HIV RNA in the saliva of all BLT mice evaluated following oral transmission of cell-associated HIV (Figure 6B).

Since transmission of HIV in humans may occur in the upper GI tract, we also determined whether cell-associated HIV could infect BLT mice if they were exposed by gavage, bypassing the oral cavity. When HIV-infected cells were directly introduced into the stomach of BLT mice by gavage, we observed 100% transmission (Figure 6C). These results indicate that the mucosal surfaces distal to the esophagus are susceptible to infection with cell-associated HIV and that transmission of cell-associated HIV can occur in the oral cavity and/or upper GI tract.

### Effect of human breast milk on oral HIV transmission

**Figure 7:**
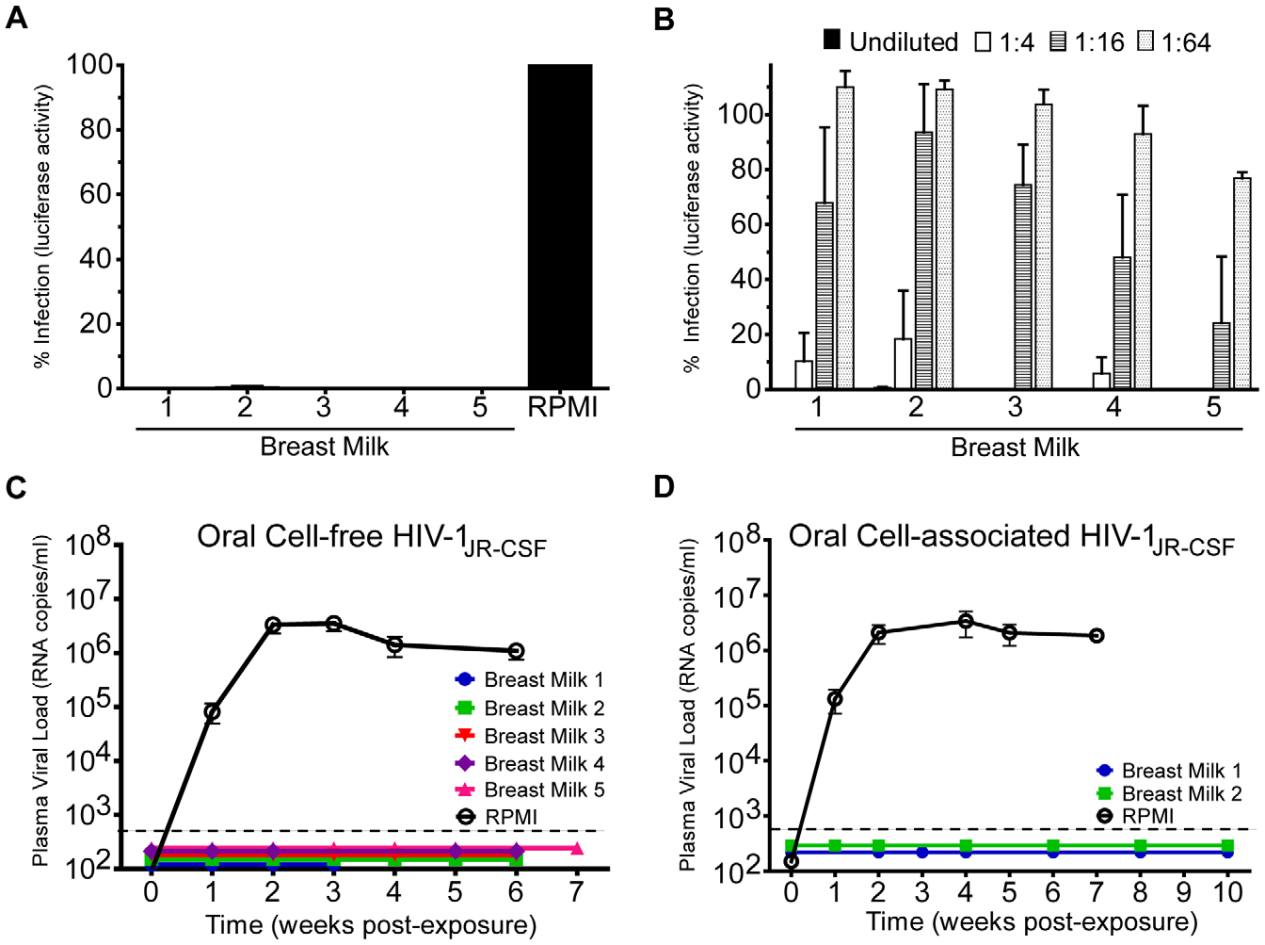
Oral Transmission of HIV in the presence of human breast milk. (A) *In vitro* inhibitory activity of human breast milk from five different donors on infection with cell-free HIV. Infection was normalized to that of cells infected with virus in the absence of milk (positive control). Note the strong inhibition observed for all 5 human breast milk samples tested. (B) Concentration dependence of the *in vitro* inhibitory activity of human breast milk on infection with cell-free HIV. HIV infection was normalized to that of cells infected with virus in the absence of milk (positive control) and compared to that of cells infected with virus in the presence of different amounts of whole human breast milk (at the indicated dilutions). (C and D) Human breast milk potently inhibits cell-free and cell- associated HIV transmission *in vivo*. HIV transmission was examined by exposing two groups of mice orally to cell-free (C) or cell-associated (D) HIV-1_JR-CSF_ in the presence of breast milk or RPMI medium. The peripheral blood viral load of mice was monitored weekly by real-time PCR. The mean peripheral blood viral load is shown for each exposure group. The limit of detection for the assay is illustrated with a dashed line.

Since the vast majority oral HIV transmission events occur in the context of human breast milk, we next investigated the impact of human breast milk on oral transmission of HIV in BLT mice. We began by evaluating the ability of whole breast milk obtained from five HIV-negative lactating women to inhibit *in vitro* HIV-1_JR-CSF_ infection. In all cases, consistent with previous reports, whole breast milk had a complete inhibitory effect on virus infectivity (Figure 7A). We also observed that the ability of each breast milk sample to inhibit HIV infection *in vitro* was concentration dependent (Figure 7B).

We then determined whether human breast milk can inhibit oral HIV transmission. For this purpose, we exposed groups of BLT mice orally to a single dose of cell-free HIV-1_JR-CSF_ in the presence or absence of whole human breast milk. Following oral exposure, HIV transmission was evaluated as indicated above. Whereas in the absence of breast milk HIV RNA was detected in the peripheral blood plasma of all control mice exposed orally to HIV-1_JR-CSF_, no transmission events occurred when the virus was orally administered in the presence of breast milk. Specifically, we did not detect HIV RNA in the peripheral blood plasma of any mouse exposed orally to HIV-1_JR-CSF_ in the presence of human breast milk at any time point post-exposure (Figure 7C). PCR analysis for the presence of viral DNA in cells isolated from tissues harvested from BLT mice exposed orally to HIV in human breast milk failed to detect the presence of HIV DNA in any tissue analyzed. In contrast, HIV DNA was readily detected in cells isolated from the peripheral blood and tissues of all BLT mice exposed orally to virus in the absence of breast milk (Table 2). In addition, to confirm that the BLT mice exposed orally to HIV in breast milk were indeed susceptible to HIV infection, three weeks following the original oral exposure to cell-free HIV in breast milk of donor 1, these same BLT mice (mice 11–13) were re-exposed orally to the same virus in the absence of milk. Under these conditions the virus was readily transmitted (Table 2). Together, the results of these experiments highlight the potent HIV inhibitory activity of normal human breast milk and demonstrate that the *in vitro* HIV inhibitory activity of human breast milk is also capable of efficiently preventing oral transmission of cell-free HIV.

**Table 2:** Description of BLT mice used to evaluate the effect of human breast milk on oral transmission of cell-free HIV-1.

		At Time of Exposure		At Time of Harvest								
Breast	Mouse	PB Humanization		Week	Viral Load	Presence of Cell-associated Viral DNA*						
Milk	ID	%CD45^+^	%CD45^+^CD3^+^CD4^+^	Post-Exposure	(RNA copies/ml)	SPL	LN	BM	TO	LIV	LNG	PB
None	1	56	83	2	154,486	+	+	nd	**−**	nd	nd	+
	2	49	87	3	7,637,214	nd	nd	nd	nd	nd	nd	nd
	3	56	84	3	6,111,176	nd	nd	nd	nd	nd	nd	nd
	4	66	85	3	5,856,373	nd	nd	nd	nd	nd	nd	nd
	5	36	77	4	3,775,970	nd	nd	nd	nd	nd	nd	nd
	6	44	82	6	2,063,909	+	nd	+	+	+	**−**	+
	7	78	84	8	850,951	+	+	+	+	+	+	+
	8	57	82	8	741,901	+	nd	+	+	+	+	+
	9	32	69	6	827,983	+	nd	nd	+	+	+	+
	10	62	67	2	1,895,784	+	nd	+	+	+	+	nd
1a	11	72	84	3	Negative	Re-exposed orally to HIV in medium (1b)						
	12	59	76	3	Negative	Re-exposed orally to HIV in medium (1b)						
	13	44	86	3	Negative	Re-exposed orally to HIV in medium (1b)						
1b	11	76	84	1	Negative	nd	nd	**−**	nd	nd	nd	**−**
	12	71	75	4	1,490,203	+	+	+	+	+	+	+
	13	50	79	8	3,133,182	nd	nd	nd	nd	nd	nd	+
2	14	60	85	6	Negative	**−**	**−**	**−**	**−**	**−**	**−**	**−**
	15	49	67	6	Negative	**−**	−	**−**	**−**	nd	nd	**−**
	16	64	72	6	Negative	**−**	**−**	**−**	**−**	nd	nd	**−**
3	17	31	80	4	Negative	**−**	**−**	**−**	**−**	**−**	**−**	**−**
	18	32	83	6	Negative	**−**	**−**	**−**	**−**	**−**	**−**	nd
	19	40	82	3	Negative	**−**	**−**	**−**	**−**	**−**	**−**	**−**
	20	47	82	6	Negative	**−**	**−**	**−**	**−**	**−**	**−**	**−**
4	21	56	79	3	Negative	nd	**−**	**−**	**−**	**−**	**−**	nd
	22	83	87	6	Negative	**−**	**−**	**−**	**−**	**−**	**−**	**−**
	23	68	78	6	Negative	**−**	**−**	**−**	**−**	**−**	**−**	**−**
	24	51	84	6	Negative	**−**	**−**	**−**	**−**	**−**	**−**	**−**
5	25	80	87	7	Negative	**−**	**−**	**−**	**−**	nd	nd	**−**
	26	55	85	6	Negative	**−**	**−**	**−**	**−**	**−**	**−**	**−**
	27	53	89	7	Negative	**−**	**−**	**−**	**−**	nd	nd	**−**
	28	76	73	6	Negative	**−**	**−**	**−**	**−**	nd	nd	**−**

* Real-time PCR results representative of DNA extracted from 5×10^4^–1.6×10^6^ cells or 15–50 ul blood. The assay limit of detection is 10 copies. SPL = spleen, LN = lymph node, BM = bone marrow, TO = thymic organoid, LIV = liver, LNG = lung and PB = peripheral blood. The results are indicated as follows: (+) positive for HIV DNA, (−) negative for HIV DNA, (nd) not determined.

Once we established that whole human breast milk can inhibit oral transmission of cell-free HIV in BLT mice, we proceeded to determine the potential inhibitory effect of human breast milk on oral transmission of cell-associated HIV given that both cell-free and cell-associated HIV can be detected in the breast milk of HIV-infected women [15], [43]–[45]. We evaluated the impact of human breast milk on oral transmission of cell-associated HIV using breast milk that inhibited oral transmission of cell-free HIV-1_JR-CSF_.In stark contrast to the robust transmission of cell-associated HIV after oral exposure in the absence of breast milk, cell-associated HIV was not capable of establishing a productive infection when oral exposures occurred in the presence of breast milk. Specifically, we did not detect HIV RNA in the plasma of BLT mice that were exposed orally to HIV-infected cells re-suspended in the breast milk of donors 1 or 2 at any time point post-exposure (Figure 7D). Furthermore, the lack of HIV transmission was confirmed at necropsy when no HIV DNA was detected in the peripheral blood or tissues of any BLT mouse exposed to cell-associated HIV in the presence of human breast milk (Table 3). Collectively, these data demonstrate that BLT mice are susceptible to oral transmission of cell-associated HIV and that human breast milk possesses innate factors that can potently inhibit oral transmission of both cell-free and cell-associated HIV.

**Table 3:** Description of BLT mice used to evaluate the effect of human breast milk on oral transmission of cell-associated HIV-1.

		At Time of Exposure		At Time of Harvest								
Breast	Mouse	PB Humanization		Week	Viral Load	Presence of Cell-associated Viral DNA*						
Milk	ID	%CD45^+^	%CD45^+^CD3^+^CD4^+^	Post-Exposure	(RNA copies/ml)	SPL	LN	BM	TO	LIV	LNG	PB
None	29	52	77	8	3,337,726	+	+	+	+	+	+	+
	30	53	84	11	436,747	+	+	+	+	+	+	+
	31	72	84	3	2,840,509	+	+	+	+	+	+	+
	32	56	80	3	2,823,067	+	+	+	+	+	+	+
	33	77	80	7	2,148,417	+	nd	+	+	+	+	+
	34	78	82	7	1,924,391	+	nd	+	+	+	+	+
	35	39	81	5	3,715,198	+	+	+	+	+	+	+
1	36	68	80	3	Negative	**−**	**−**	**−**	**−**	**−**	**−**	**−**
	37	23	82	10	Negative	**−**	**−**	**−**	**−**	**−**	**−**	**−**
	38	62	77	6	Negative	**−**	**−**	**−**	**−**	**−**	**−**	**−**
	39	37	79	6	Negative	**−**	**−**	**−**	**−**	**−**	**−**	**−**
2	40	24	74	10	Negative	**−**	**−**	**−**	**−**	**−**	**−**	**−**
	41	42	87	10	Negative	**−**	**−**	**−**	**−**	**−**	**−**	**−**
	42	76	77	8	Negative	**−**	**−**	**−**	**−**	**−**	**−**	**−**
	43	46	77	3	Negative	**−**	**−**	nd	**−**	**−**	**−**	nd

* Real-time PCR results representative of DNA extracted from 1×10^5^–1×10^6^ cells, snap frozen tissue or 15 ul blood. The assay limit of detection is 10 copies. SPL = spleen, LN = lymph node, BM = bone marrow, TO = thymic organoid, LIV = liver, LNG = lung and PB = peripheral blood. The results are indicated as follows: (+) positive for HIV DNA, (−) negative for HIV DNA, (nd) not determined.

### Prevention of oral HIV transmission by antiretroviral pre-exposure prophylaxis

Previously our laboratory demonstrated that BLT mice administered systemic pre-exposure prophylaxis (PrEP) of emtricitabine (FTC)/tenofovir disoproxil fumarate (TDF) were efficiently protected from HIV-1 infection following intravenous, rectal and vaginal challenges [19], [20]. To assess whether systemic FTC/TDF PrEP can also prevent oral transmission of HIV-1 in BLT mice, we administered systemic FTC/TDF to BLT mice once daily for 7 days and exposed mice orally to cell-free HIV-1_JR-CSF_ 3 hours after the third administration of antiretrovirals as previously described [19], [20]. Subsequently, HIV transmission was monitored in peripheral blood by measuring the plasma viral load. In addition, at necropsy we utilized real-time PCR to evaluate whether HIV DNA was present in the peripheral blood or any tissues harvested from BLT mice. No viral RNA was detected in the peripheral blood plasma of any BLT mouse receiving the 7-day course of systemic FTC/TDF at any time point post-exposure. We also did not detect the presence of HIV DNA in their peripheral blood or in any of their tissues at necropsy confirming lack of infection (Table 4). These results demonstrate that oral transmission of HIV in BLT mice can be efficiently prevented by the administration of systemic FTC/TDF and serve as a proof of concept for future studies aimed at evaluating the efficacy of novel HAART strategies.

**Table 4:** Oral transmission of cell-free HIV-1 in BLT mice following systemic FTC/TDF PrEP.

	At Time of Exposure		At Time of Harvest								
Mouse	PB Humanization		Week	Number of Negative	Presence of Cell-associated Viral DNA*						
ID	%CD45^+^	%CD45^+^CD3^+^CD4^+^	Post-Exposure	Plasma Viral Loads	SPL	LN	BM	TO	LIV	LNG	PB
44	59	84	11	7 of 7	−	−	−	−	−	−	−
45	76	87	11	7 of 7	−	−	−	−	−	−	−
46	74	85	11	7 of 7	−	−	−	−	−	−	−
47	82	89	10	6 of 6	−	−	−	−	−	−	−
48	71	89	10	6 of 6	−	−	−	−	−	−	−

* Real-time PCR results representative of DNA extracted from 8.75×10^5^–1×10^6^ cells or 15 ul blood. The assay limit of detection is 10 copies. SPL = spleen, LN = lymph node, BM = bone marrow, TO = thymic organoid, LIV = liver, LNG = lung and PB = peripheral blood. The results are indicated as follows: (+) positive for HIV DNA, (−) negative for HIV DNA, (nd) not determined.

## Discussion

Avoidance of breastfeeding by HIV seropositive mothers in resource limited settings where prophylaxis is not available reduces the risk of HIV transmission but does not increase the overall survival of their children; breastfeeding protects these children from infections that result in diarrhea, pneumonia and sepsis [14]. In this study, we first established that human hematopoietic cells generated *in situ* are capable of repopulating the oral cavity and upper GI tract of humanized BLT mice. Specifically, these important mucosal tissues of BLT mice are repopulated with the types of human cells that have been identified to be important for mucosal HIV transmission (i.e. dendritic cells, macrophages and CD4^+^ T cells). Our results demonstrate that the presence of these human cells renders BLT mice susceptible to oral transmission of cell-free and cell-associated HIV. Oral transmission of HIV results in systemic infection of lymphoid and non-lymphoid tissues that is characterized by a gradual decline of CD4^+^ T cells in peripheral blood. In addition, infection of the oral cavity results in virus shedding into saliva, recapitulating the human condition [27], [28]. Our data also offers the first *in vivo* demonstration that human breast milk can inhibit oral transmission of cell-free and cell-associated HIV. Furthermore, oral transmission of HIV can be prevented with systemic FTC/TDF PrEP.

Previous studies utilizing NOD/SCID and NOD/SCID/β_2_m^−/−^ mice reconstituted with human peripheral blood leukocytes (hu-PBL mice) failed to demonstrate oral transmission of cell-free HIV following an oral exposure to CXCR4 and CCR5 tropic strains, including HIV-1_JR-CSF_ [46]. Therefore, our work represents a significant advance since we demonstrated, for the first time, highly reproducible oral transmission of multiple HIV strains in BLT mice. Specifically, in this study we demonstrate oral transmission of the well characterized CCR5-tropic HIV-1 isolate JR-CSF and of several T/F viruses. Our data revealed that the efficiency of oral transmission varied among T/F viruses, suggesting that intrinsic properties of these viruses may contribute to their transmission *in vivo*. Recent studies of mother-infant transmission pairs indicate that the replicative fitness as well as the length of the variable loop and number of n-linked glycosylation sites in the envelope protein may influence which maternal variant(s) are preferentially transmitted [47]–[49]. Our results indicate that BLT mice could be used to study the molecular and biological properties of HIV strains that provide a selective advantage for oral transmission during breastfeeding. A comparison of viruses transmitted at early, mid and late stages of lactation would indicate if the composition of breast milk, which changes during lactation, influences which viruses are preferentially transmitted.

During breastfeeding, HIV is transmitted from mother-to-child in the context of human breast milk. In the absence of antiretroviral therapy, the breast milk of most HIV-infected women possesses cell-free and/or cell-associated HIV [15]–[17]. Although elevated levels of both cell-free and cell-associated HIV in the breast milk of HIV-infected mothers have been associated with an increased risk for HIV transmission during breastfeeding [15]–[17], it is currently not known if both cell-free and cell-associated HIV are transmitted to infants. Studies illustrating that HAART administered to HIV-infected mothers during pregnancy or post-partum significantly decreases the amount of cell-free but not cell-associated HIV in breast milk [50], [51], combined with reports demonstrating that maternal HAART significantly decreases but does not eliminate HIV transmission during breastfeeding, suggest that both cell-free and cell-associated HIV may be transmitted [52], [53]. As the importance of cell-associated HIV in breast milk transmission becomes increasingly more appreciated, antiretroviral drugs and preventative strategies may be needed that reduce the burden of HIV-infected cells in the breast milk of HIV-infected women and/or directly inhibit transmission of cell-associated HIV in infants during breastfeeding. Our data demonstrating oral transmission of both cell-free and cell-associated HIV is of high relevance since it will make possible the future evaluation of novel prophylactic strategies aimed at preventing oral transmission of both cell-free and cell-associated HIV. However, even though infected cells were used for the exposures, we cannot rule out the possibility that actual transmission across the mucosal surface could occur with cell-free virions released from infected cells.

Paradoxically, although breastfeeding can be attributed to a significant number of HIV infections in children, breast milk has been shown to potently inhibit HIV infectivity and to possess several innate factors with *in vitro* anti-HIV inhibitory activity [4]–[12]. Our results offer the first *in vivo* evidence that human breast milk can strongly inhibit oral transmission of both cell-free and cell-associated HIV.

The ability of human breast milk to inhibit cell-associated HIV transmission in BLT mice is in contrast to *in vitro* studies suggesting that milk does not inhibit cell-associated infection [54]. This apparent discrepancy may be explained by the use of whole human breast milk for our experiments. Specifically, the *in vitro* experiments comparing breast milk inhibition of cell-free and cell-associated HIV infection utilized the skim milk fraction of breast milk. Further *in vitro* analysis comparing inhibition of cell-free HIV infection in the presence of whole breast milk or the skim milk fraction will be needed to address this issue. However, since most children at risk of HIV infection via breast milk do not receive skim milk, the potential relevance of this *in vitro* observation may be questionable. Nevertheless, inhibitory factors present in breast milk may differ in their ability to inhibit cell-free versus cell-associated HIV infection. While the skim milk fraction of human breast milk possesses proteins with HIV inhibitory activity (i.e. mucin, lactoferrin, bile salt-stimulated lipase and secretory leukocyte protease inhibitor [SLPI]) [4]–[12], the lipid fraction may contain additional factors that can inhibit transmission of cell-free and/or cell-associated HIV. For example, increased concentrations of certain long-chain polyunsaturated fatty acids (LCPUFAs) in breast milk are associated with a decreased risk of HIV breastfeeding transmission [55]. Although their ability to inhibit cell-free versus cell-associated HIV infection has not been experimentally tested to our knowledge, it has been hypothesized that LCPUFAs may inhibit HIV infection by inactivating the virus' envelope, suppressing the release of HIV virions from the host cell membrane and/or enhancing the viability of infected CD4^+^ T cells [56]. All together, our results highlight the protective role of human breast milk against HIV transmission and suggest that components in both the skim milk and lipid fractions may contribute to its HIV inhibitory activity.

Despite ingesting liters of breast milk over a span of several months to years, the majority of infants born to HIV-infected HAART naïve women (∼85%) do not acquire HIV during breastfeeding [14]. This observation is in agreement with our data demonstrating the potent *in vivo* inhibitory activity of human breast milk on oral transmission of both cell-free and cell-associated HIV following a single oral exposure. Oral transmission of HIV in the presence of human breast milk may require multiple exposures over time. Furthermore, although increased levels of HIV in breast milk have been associated with an increased risk for HIV transmission during breastfeeding, several other maternal and infant factors have been associated with breastfeeding transmission. Additional maternal factors include seroconversion during lactation, CD4^+^ T cells counts below 500 cells per mm^3^, poor breast health (mastitis, nipple bleeding, etc), and decreased levels of alpha-defensins in breast milk. Infants that receive both breast milk and other food (mixed-feeding) are also more susceptible to HIV transmission during breastfeeding as are infants with oral thrush and decreased levels of salivary SLPI [reviewed in [14]]. One remaining question is, therefore, whether or not there are differences in the babies or mothers or in the breast milk in the cases where mother-to-child transmission does occur. In the future, it will be important to compare the inhibitory activity of breast milk obtained from HIV-infected mothers who transmit HIV to that of HIV-infected mothers that do not transmit HIV *in vivo*. In addition, *in vivo* experiments evaluating transmission in the presence of saliva obtained from infants with low and high levels of salivary SLPI will help assess the contribution of the inhibitory activity of infant salivary SLPI on oral transmission of cell-free and cell-associated HIV.

Collectively, our results demonstrate that BLT mice are an attractive small animal model that can be utilized to study key aspects of oral HIV transmission and to test the efficacy of HIV vaccines, antiretroviral therapies and other preventative measures aimed at reducing mother-to-child transmission of HIV during breastfeeding. In addition, our data demonstrating the presence of human immune cells in the oral cavity and GI tract of BLT mice indicate that BLT mice may be utilized to study other human pathogens that are transmitted orally and/or infect these tissues (i.e. HCMV and EBV) and to answer fundamental questions about human oral and gastrointestinal immunity.

## Materials and Methods

### Ethics statement

All animal experiments were conducted following NIH guidelines for housing and care of laboratory animals and in accordance with The University of North Carolina at Chapel Hill (UNC-Chapel Hill) regulations after review and approval by the UNC-Chapel Hill Institutional Animal Care and Use Committee (permit number 09-158).

### Preparation of humanized BLT mice

Humanized BLT mice were prepared as previously described [18]–[23]. Briefly, a 1–2 mm piece of human fetal liver tissue was sandwiched between two pieces of autologous fetal thymus tissue (Advanced Bioscience Resources, Alameda, CA) under the kidney capsule of sublethally irradiated (300 cGy) 6–8 wk old NOD.Cg-*Prkdc^scid^ Il2rg^tm1Wjl^*/SzJ (NSG; The Jackson Laboratory, Bar Harbor, ME) mice. Following implantation, mice were transplanted intravenously with hematopoietic CD34^+^ stem cells isolated from autologous human fetal liver tissue. Human immune cell reconstitution was monitored in the peripheral blood of BLT mice by flow cytometry every 3–4 weeks as previously described [18]–[20], [22], [23]. Mice were maintained by the Division of Laboratory Animal Medicine under specific-pathogen free conditions at UNC-Chapel Hill.

### Immunohistochemical analyses

Tissues for IHC were harvested from BLT mice and fixed in 4% paraformaldehyde for 24 hr at 4°C, embedded in paraffin, cut into 5 µm sections and mounted onto poly-L-lysine coated glass slides. Prior to paraffin embedding, the upper head region containing the NALT was decalcified in a 0.24 M EDTA solution for 7–10 days at 4°C. Following paraffin removal, antigen retrieval (DIVA Decloaker, Biocare Medical, Concord, CA) and blocking of non-specific Ig-binding sites (Background Sniper, Biocare Medical), tissue sections were stained with primary antibodies overnight at 4°C and developed with a biotin-free HRP-polymer system (MACH3 Mouse or Rabbit HRP-Polymer Detection, Biocare Medical). All tissue sections were then counterstained with hematoxylin. Images were taken with an upright Nikon Microphot SA microscope with a DXM 1200 color camera and the white balance and brightness adjusted in Adobe Photoshop CS4.

Primary antibodies directed against the following human antigens were used to verify the presence of specific human immune cell populations in the oral buccal mucosa (n = 7), submandibular salivary glands (n = 5), CLN (n = 4), NALT (n = 4), esophagus (n = 7), stomach (n = 5) and duodenum (n = 4) of BLT mice: CD3 (F7.2.38, Dako, Carpinteria, CA), CD4 (1F6, Leica, Buffalo Grove, IL and SP35, GenWay, San Diego, CA), CD8 (C8/144B, Dako), CD11c (5D11, Leica), CD20 (L26, Biocare Medical), CD45 LCA (2B11&PD7/26, Dako) and CD68 (KP1, Dako). HIV-infected cells were detected with an antibody directed against HIV p24 Gag (Kal-1, Dako). As a control, tissue sections were stained with the following isotype control antibodies: mouse IgG1k (Dako), mouse IgG2a (Dako) and rabbit Ig (Dako).

### Exposure of humanized BLT mice to cell-free HIV-1

Stocks of HIV-1_JR-CSF_, HIV-1_RHPA_, HIV-1_CH040_, and HIV-1_CH077_ [40]–[42], [57] were prepared and titrated as previously described [19]–[23]. Oral inoculations of mice were performed by placing anesthetized BLT mice on their backs and instilling virus directly into their mouth. To ensure that all surfaces of the oral cavity were exposed to virus, initial oral exposure experiments were performed with 2.82×10^6^ TCIU of HIV-1_JR-CSF_ diluted in RPMI medium or normal human breast milk (Innovative Research, Novi, MI) to a final volume of 50 µl. Once all surfaces of the oral cavity (palate, tongue, gums and epithelium lining the cheek, lip and underside of the tongue) were exposed to virus, the excess virus was pipetted out of the oral cavity and the volume measured to determine the actual inoculum (approximately 0.9–1.5×10^6^ TCIU of HIV-1_JR-CSF_). Mice were held in place for 5 minutes to ensure retention of the virus. Once mice recovered from anesthesia, they were permitted immediate access to food and water. Subsequent oral exposures to HIV-1_JR-CSF_ were performed using a total volume of 20 µl and 1.4×10^6^ TCIU of virus. BLT mice exposed orally to HIV-1 T/F viruses were administered 6×10^5^ TCIU of HIV-1_RHPA_, HIV-1_CH040_, HIV-1_CH077_ or HIV-1_JR-CSF_ (positive control) in 20 µl RPMI medium. To assess the efficacy of systemic FTC/TDF PrEP on oral transmission of cell free HIV in BLT mice, mice were administered FTC/TDF (Gilead, Foster City, CA) intraperitonealy (3.5 mg and 5.2 mg, respectively) once daily for seven consecutive days. Three hours after the third administration of FTC/TDF, BLT mice were exposed orally to 1.4×10^6^ TCIU cell-free HIV-1_JR-CSF_.

HIV was directly introduced into the stomach of BLT mice by gavage. Gavages were performed without anesthesia by threading a feeding needle with a ball tip into the mouth and down the esophagus of BLT mice until it reached the stomach. Next, using a total volume of 100 µl, 1.4×10^6^ TCIU of HIV-1_JR-CSF_ in RPMI medium was directly introduced into the stomach.

### Exposure of humanized BLT mice to cell-associated HIV-1

In order to generate cell-associated HIV, human PBMCs were infected *in vitro* with HIV-1_JR-CSF_. PBMCs were cultured with 5 µg/ml PHA and 20 U/ml IL-2 in IMDM medium containing 10% FBS and 1% pen/strep for 3 days prior to infection. PBMCs were then infected at a MOI of 0.1 with HIV-1_JR-CSF_ by a 2 hr spin infection (1500×g for 2 hr at 25°C). Next, PBMCs were washed three times with Dulbecco's PBS and re-suspended in IMDM medium containing 10% FBS and 1% pen/strep. Three to four days post-infection, the percent of HIV-infected cells was determined by intracellular staining for HIV p24 Gag (KC57-FITC, Beckman Coulter, Brea, CA) using the Fix & Perm kit (Invitrogen, Grand Island, NY). BLT mice were exposed orally to 3.75×10^5^ HIV p24 Gag^+^ PBMCs (up to 1.9×10^7^ total PBMCs) in RPMI medium or human breast milk using a total volume of 20 µl as described for oral exposures of BLT mice to cell-free HIV. Gavages were performed as described above with 3.75×10^5^ HIV p24 Gag^+^ HIV-1_JR-CSF_ infected PBMCs (up to 1.6×10^7^ total PBMCs) in RPMI medium using a total volume of 100 µl.

### Analysis of HIV-1 infection in humanized BLT mice

Infection of humanized BLT mice with HIV was monitored in peripheral blood plasma by measuring the levels of viral RNA with a one-step reverse transcriptase real-time PCR assay using the primers 5′-CATGTTTTCAGCATTATCAGAAGGA-3′ and 5′-TGCTTGATGTCCCCCCACT-3′ and the MGB-probe 5′-FAM (6-carboxyfluorescein)-CCACCCCACAAGATTTAAACACCATGCTAA-Q (6-carboxytetramehtylrhodamine)-3′ [58]; assay sensitivity of 400 HIV RNA copies/ml [22]. The percentage of human CD4^+^ T cells in peripheral blood of BLT mice pre-exposure (0–2 weeks prior to exposure) and post-exposure were determined by flow cytometry. Saliva was collected following an intraperitoneal injection with pilocarpine (100 µl at 1 mg/ml) to stimulate saliva production and the levels of HIV RNA measured using the real-time PCR assay described above. The presence of viral DNA in tissues and peripheral blood collected from BLT mice was determined by real-time PCR analysis of DNA extracted from 5×10^4^–4×10^6^ cells or 15–50 µl peripheral blood cells as previously described; assay sensitivity of 10 DNA copies [19], [20], [22]. As a control, all samples were tested for the presence of human gamma globulin DNA by real-time PCR.

### *In vitro* assessment of the inhibitory activity of human breast milk on cell-free HIV infection

Breast milk obtained from five HIV-negative women (Innovative Research) at unknown time points during lactation was used to test the *in vitro* inhibitory activity of human breast milk on cell-free HIV infection. TZM-bl cells were plated in a white, opaque 96-well plate the day prior to infection at 1×10^5^ cells per well. Human breast milk or RPMI medium (5 µl) was incubated with 5 µl of HIV-1_JR-CSF_ (6×10^4^ TCIU) for 10 min at RT. Since breast milk was found to be toxic to TZM-bl cells, the breast milk-virus mixture was diluted 1∶400 with DMEM medium containing 10% FBS, 25 mM HEPES, 1% pen/strep and 40 µg/ml DEAE Dextran and then vortexed briefly before adding it to the TZM-bl cells (3×10^3^ TCIU HIV-1_JR-CSF_ per well). This dilution of milk was found to be non-toxic to TZM-bl cells (data not shown). 48 hours post-infection, the medium was removed and 100 µl of One-Glo reagent (Promega, Madison, WI) supplemented with Triton X-100 (final concentration of 0.01%) was added to inactivate virus and to allow for the measurement of luciferase activity. Luciferase was measured with a luminometer and the results normalized to the luciferase activity of cells infected with virus incubated with plain RPMI medium. For experiments assessing the inhibitory activity of serial dilutions of whole breast milk on HIV infection, breast milk was diluted with plain RPMI medium prior to incubation with virus. All experiments were performed in triplicate.

### Statistical analyses

All statistical analyses were performed in Prism, version 5 (Graph pad, La Jolla, CA). We used a two-tailed Mann-Whitney U test to compare the percentages of CD4^+^ T cells in peripheral blood pre- and post-exposure.

## Supporting Information

Figure S1 **Human reconstitution of the GE junction in BLT mice.** A portion of the stomach possessing the GE junction was harvested from BLT mice for immunohistochemical analysis to determine the presence of HIV target cells. The tissues harvested were stained with the appropriate antibodies to verify the presence of human leukocytes (CD45^+^) including dendritic cells (CD11c^+^), macrophages (CD68^+^) and T cells (CD3^+^), specifically, CD4^+^ T cells (CD4^+^). Positive cells appear brown. Scale bars = 100 µm.
(TIF)
