# Supplementary figures and images for: Human Breast Milk and Antiretrovirals Dramatically Reduce Oral HIV-1 Transmission in BLT Humanized Mice
Source: PLoS Pathog. 2012 Jun 14;8(6):e1002732. doi: 10.1371/journal.ppat.1002732 (PMC3380612; doi:10.1371/journal.ppat.1002732)

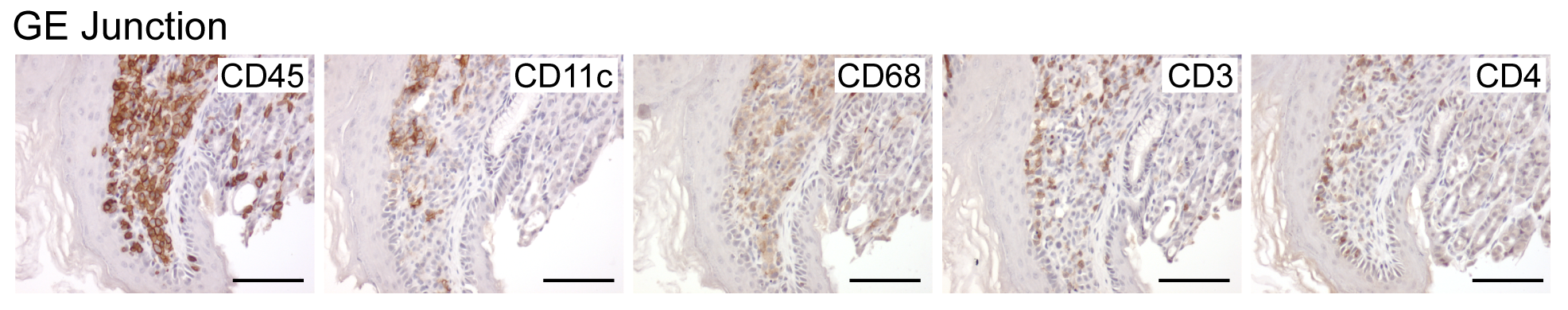

Supplement: Figure S1 — Human reconstitution of the GE junction in BLT mice. A portion of the stomach possessing the GE junction was harvested from BLT mice for immunohistochemical analysis to determine the presence of HIV target cells. The tissues harvested were stained with the appropriate antibodies to verify the presence of human leukocytes (CD45+) including dendritic cells (CD11c+), macrophages (CD68+) and T cells (CD3+), specifically, CD4+ T cells (CD4+). Positive cells appear brown. Scale bars = 100 µm. (TIF) [file ppat.1002732.s001.tif]
